# Feasibility and Safety of Liberal Fluid Fasting in an Orthogeriatric Department: A Prospective Before-and-After Cohort Study

**DOI:** 10.3390/jcm14155477

**Published:** 2025-08-04

**Authors:** Thomas Saller, Janine Allmendinger, Patricia Knabe, Max Knabe, Lina Lenninger, Anne-Marie Just, Denise Seidenspinner, Boris Holzapfel, Carl Neuerburg, Roland Tomasi

**Affiliations:** 1 Department of Anaesthesiology, LMU Munich University Hospital Munich, 81377 Munich, Germanypatricia.knabe@med.uni-muenchen.de (P.K.);; 2 Max Planck Institute for the Study of Crime, Security and Law, 79100 Freiburg im Breisgau, Germany; 3 Clinical Nursing Research and Quality Management Unit, LMU Munich University Hospital, 81377 Munich, Germany; 4 Department of Orthopaedic and Trauma Surgery, Musculoskeletal University Center Munich (MUM), LMU Munich University Hospital, 81377 Munich, Germany

**Keywords:** preoperative, fasting, general anesthesia, ultrasound, elderly, complications

## Abstract

**Background:** The rationale for strict fluid fasting for pediatric and adult patients has been questioned recently. Point-of-care tools for the evaluation of gastric content have evolved over time, often using gastric ultrasound. Usually, the gastric antral cross-sectional area (CSA) is determined. A liberal fluid fasting regimen, that is, ingestion of liquid fluids until the call for theatre, does not delay gastric emptying compared to midnight fasting, as evaluated with gastric ultrasound. Anesthesia is safe, and no adverse events result from a liberal regimen. **Methods:** The ethics committee of LMU Munich approved the study (21-0903). Liberal fluid fasting in a geriatric orthopedic surgery department (LFFgertrud) is a sub-study within a project investigating perioperative neurocognitive disorders (Study Registration: DRKS00026801). After obtaining informed consent from 134 geriatric patients 70 years or older, we investigated the gastric antral cross-sectional area (CSA) prior to and postimplementation of liberal fluid management, respectively. **Results:** After the implementation of liberal fluid fasting, fasting times for solid food and liquids decreased from 8.8 (±5.5) to 1.8 (±1.8) hours (*p* < 0.0001). In 39 patients where CSA was obtained, a slight increase in fluid was encountered. No critical amount of gastric content was observed, and no adverse events occurred. **Conclusions:** A liberal fluid fasting concept was safe even for comorbid elderly patients in orthopedic surgery. Applying a gastric ultrasound may be helpful to increase safety. According to the incidence of complications encountered in our study, it seems indispensable.

## 1. Introduction

Back in 1946, American anesthesiologist Curtis L. Mendelson first described a syndrome of aspiration of stomach contents into the lungs during obstetric anesthesia [1]. Since then, conservative regimens have been adhered to by clinicians to rule out pulmonary aspiration and regurgitation. The Practice Guidelines for Preoperative Fasting and the Use of Pharmacologic Agents to Reduce the Risk of Pulmonary Aspiration of The American Society of Anesthesiologists (ASA) were updated recently [2]. The authors recommend encouraging adult patients with a low risk of aspiration to drink up to 400 mL of carbohydrate-containing clear liquids two hours before anesthesia.

No aspiration was reported after either fasting or drinking carbohydrate-containing clear liquids in several dozen studies, as well as for vomiting, residual gastric volume, and gastric pH after fasting or drinking carbohydrate-containing clear liquids (moderate strength of evidence) [3,4,5]. Other societies, e.g., the European Society of Anaesthesiology and Intensive Care, adhere to these guidelines [6]. An update on fasting guidelines is in preparation.

However, these recommendations are poorly implemented by clinicians in order not to cause harm. Prospective assessment of real fasting times unveiled intervals between 11 and 19 h for liquid and solid food, respectively [7]. For trauma surgery in middle-aged patients, a median (interquartile range) preoperative fasting time for liquids and solids was reported 8 (5.2–12.9) and 19 (15.7–22) hours, respectively [8,9]. As longer fasting time is associated with patient discomfort, hypotension after induction, complications, and delirium, recently discussions about liberal fluid fasting have emerged [10]. For children, the fluid fasting time was reduced to two hours preoperatively by some of the anesthesiologic societies [11], while others hesitate due to little evidence.

Although there is weak evidence that liberal fluid fasting does not delay gastric emptying compared to more restrictive fasting regimes, it is supposed to be safe for younger and healthy patients [12]. Nevertheless, a specific cut-off for a harmful amount of gastric content is unknown. To elucidate this cut-off, gastric ultrasound was introduced as a point-of-care examination before the induction of anesthesia [13] and mathematical models for the calculation of gastric volume were proposed [14].

Furthermore, neither the effects of liberal fluid fasting on gastric content nor the implementation of such a concept has been studied yet in elderly patients.

For this study, we hypothesized, first, to reduce preoperative fasting time in older adults with a liberal fluid fasting initiative and secondly, to assess cross-sectional gastric area as a possible safety measure.

## 2. Materials and Methods

We designed a prospective, observational study to study the neurocognitive effects of the implementation of a liberal fluid fasting (LFF) concept on four general wards at the LMU University Hospital, Munich, an academic tertiary hospital in southern Germany. Patients 70 years or older who were prone to a planned, at least 60 min-long surgical procedure in orthopedic or trauma surgery were invited to participate. Inability to give informed consent or incident delirium, inadequate ability to communicate in the German language, parenteral nutrition, pre-existing or acute gastrointestinal conditions, cardiac or intracranial surgery, or a palliative situation were exclusion criteria. The primary outcome was the incidence of delirium. The CSA results prior to and following the implementation of a liberal fluid fasting concept and major postoperative complications were the secondary outcomes. However, in this manuscript, we focus on the association of fasting times with the results of the gastric ultrasound assessments. After obtaining informed consent, the Mini-Cog, a quick screening test for short-term memory and visual–spatial ability, and the Montreal Cognitive Assessment (MoCA) were performed. Functional performance was documented using the instrumented activities in daily living (IADL), the FRAIL-Scale, and the European Quality of Life 5 Dimensions 5 Level Version (EQ-5D-5L) to document overall health in five categories as mobility, self-care, usual activities, pain and discomfort, and anxiety and depression. The latter was complemented by the geriatric depression scale (GDS). The nutritional status was assessed with the mini nutritional assessment long form (MNA-LF). Patients were also asked about their fluid intake, including the amount of fluid consumed over the last three days; thirst, dryness of the skin and mucous membranes were to be rated on a four-step Likert scale, and urine volume and color on a three-step Likert scale.

As of standard, upon arrival in the preoperative holding area, patients were informed by the nurse about their last meal and drink. Hunger and thirst were rated on a 6-item Likert scale by the patient. After surgery, delirium and major complications were assessed daily for five postoperative days. Three, six, and twelve months after surgery, the SBT, IQCODE, the Barthel index, and EQ-5D-5L were requested during a phone call. Results will be published after completion of the study.

The ethics committee of the LMU Munich advised and affirmed the study (21-0903). The study was registered at the German trials register in advance (DRKS00026801). Data was analyzed using IBM SPSS 27.0 (IBM Inc., Armonk, NY, USA).

Intervention. As standard, patients in cohort 1 were told conservative fasting guidelines to strictly fast for at least 6 h before surgery, not to smoke or chew gum, usually beginning from midnight, and to drink small sips of water until 6 o’clock in the morning. For taking prescribed medication, a small sip of water was allowed at any time, consistent with international guidelines.

In a predefined fashion (ward by ward), the implementation of liberal fluid fasting was delivered through verbal instructions, information leaflets for ward nurses and doctors, and labels attached to the patients’ beds instructing them to drink until 6 am if they were scheduled for surgery first, or until they were called for theatre. Patients in cohort 2 were encouraged to have a normal meal the evening before surgery and to drink plenty of water at their discretion, irrespective of any practice they used to know. After their call to the theatre, no more drinking was allowed.

In both cohorts, immediately before induction of anesthesia, one of two trained and certified investigators (RT and TS) performed a gastric ultrasound and documented gastric antral cross-sectional area (CSA). Gastric volume was then calculated by the equation published by Perlas et al.: gastric volume [mL] = 27 + (14.6 × CSA [cm^−2^]) − (1.28 × age [years]). Negative values represent no gastric content. For security reasons to prevent possible aspiration, the finding of a grade 2 stomach was considered potentially harmful, and special measures (rapid sequence induction, postponement) were taken. The perioperative anesthetic course was documented in the case report form.

## 3. Results

**Figure 1:**
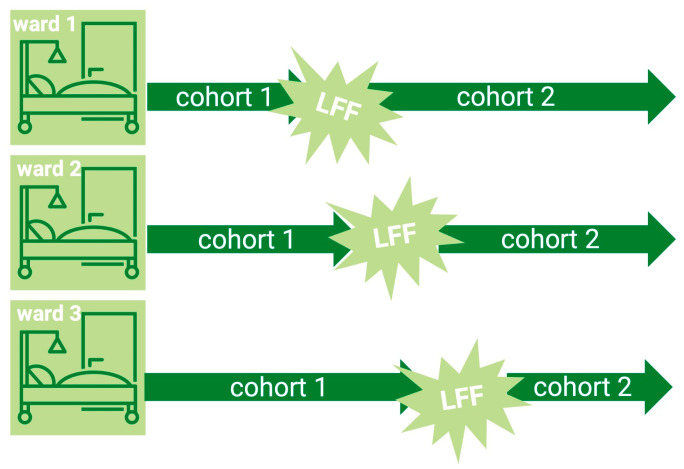
Visualization of the timeline of how liberal fluid fasting was introduced on three orthogeriatric wards. For cohort 1, the usual fasting regimen was observed. After the introduction of liberal fluid fasting (cohort 2), patients were invited to drink water until their transport to the preoperative holding area.

A total of 134 patients were included in the study. Sixty-four patients followed a conservative fasting regimen, and 70 followed a liberal fluid regimen. The study flow chart is depicted in Figure 1. Demographic comorbidities and clinical data did not differ between the two cohorts (see Table 1). Fasting times for solids as well as for liquids were reduced significantly by the intervention, and patients’ well-being was improved (see Table 2). Perioperative and postoperative measures were not affected by the intervention.

CSA assessment was feasible for all the patients examined. However, due to organizational challenges, CSA could be obtained only in 39 (29%) of the patients of the cohort. The reasons were workload, postponed surgery, or intervention outside working hours. A sample size calculation was performed in advance, resulting in a minimum group size of 116 patients. Based on a sensitivity analysis conducted post hoc, as we could only carry out CSA for 29% of the sample, the minimum detectable effect size compatible with 80% power and level of significance α = 0.05 was substantially larger (d = 0.93) than the effects observed in our data. CSA showed a Cohen’s d of 0.16, and gastric content a Cohen’s d of −0.07. This indicates that the statistical power of the study was likely insufficient to reliably detect small to moderate effects. Although the initially planned sample size was not achieved, the results provide preliminary evidence and should be interpreted with caution due to limited statistical power.

**Figure 2:**
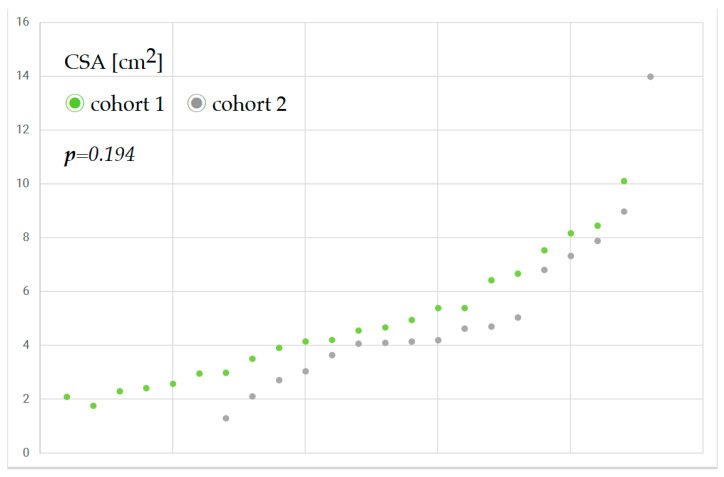
Antral cross-sectional area (CSA) of patients before (grey) and after (green) the implementation of a liberal fluid fasting concept on three orthogeriatric wards.

Figure 2 shows the antral cross-sectional area of patients before and after the implementation of the liberal fluid fasting concept and reveals only a slight increase in gastric content.

**Figure 3:**
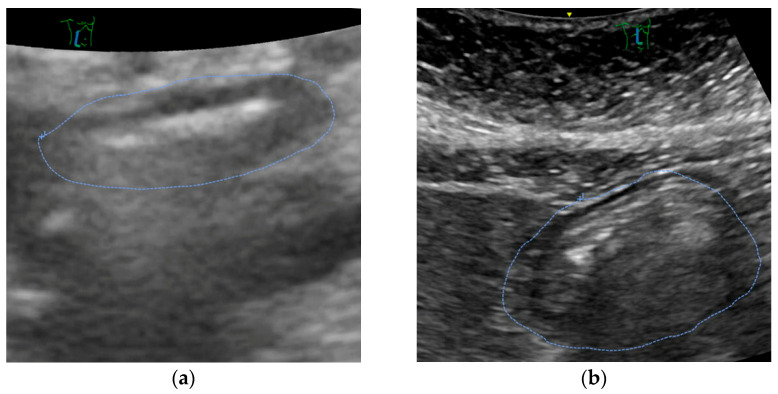
Screenshot of a representative gastric ultrasound exam performed in a patient (**a**) with an empty stomach (CSA 2.98 cm^−2^ corresponding to a calculated gastral volume of 70.5 mL) and (**b**) with a full stomach (CSA 12.0 cm^2^, 202 mL). Both patients had no liquids for more than two hours before the exam.

Mostly, ultrasound revealed an empty stomach (Figure 3a). In some patients, we found proof that tablets were taken in the morning or immediately before the call for theatre. Only in a small number of patients, remnants of clear liquids were documented (see Figure 3b). No solids were encountered.

In one patient, an atypical gastric configuration was discovered. The patient reported having a 4/5-gastric resection, an undisclosed condition at the preoperative assessment, leading to a change in anesthetic approach with a rapid sequence induction procedure.

A small number of patients in the intervention group (usually hospitalized on the morning of surgery) did not adhere to the LFF concept because they were ordered to fast by a nurse or doctor not associated with the study.

Gastric content was slightly but not relevantly increased after implementing liberal fluid fasting, with no case changing anesthetic approach due to excessive content. The number of grades 1 or 2 stomach according to Perlas was not changed. No severe adverse event was encountered. No perioperative aspiration occurred, and the rate of postoperative pneumonia was unchanged (Table 3). In contrast, urinary tract infections decreased.

## 4. Discussion

In our study, we investigated gastric content calculated by gastric antral cross-sectional area (CSA) prior to and post implementation of a liberal fluid management regimen, respectively. We found no matter of harm in offering selected elderly patients a liberal fasting approach with clear fluids until call for theatre, even if they suffered from relevant comorbidities. Fasting time was reduced significantly, and patients’ well-being was improved. This supports and complements the work of Chen et al. [4].

In one case, a gastric ultrasound encountered a possibly deleterious condition leading to a change in anesthetic management.

The method of CSA assessment before induction is restrained by the need for a trained investigator, a prerequisite that we failed even in this carefully arranged prospective study. In our case, the antral assessment was performed by two trained examiners. Measurement of the gastric antrum is highly reproducible, so observer bias is unlikely [15]. Nevertheless, despite careful planning in advance, we were unable to examine more patients. This was mainly due to organizational hurdles such as rescheduling of the operating theatre schedule, deployment of the trained sonographer in another area to support day-to-day business, lack of availability of the ultrasound machine, or uncertainty about patient participation in the study. These problems are certainly also due to the size of the hospital and the team and could possibly be better solved in a smaller setting.

In support, CSA was planned as an additional safety measure for patients with low risk for aspiration. However, as demonstrated in one patient after gastric resection, routinely administering CSA in the supine position can easily rule out large amounts of gastric content and increase safety. Especially in patients who are non-compliant with fasting rules, gastric ultrasound can contribute to increased individual patient safety [16].

Ultrasound is available in nearly every hospital and theatre today. Thus, clinicians should be encouraged to both introduce liberal fluid fasting in selected elderly patients and to ensure excessive gastric content before induction.

However, current guidelines do not yet support this approach. Although in children, fluid fasting time has been reduced, there is little evidence to support this recommendation. Further and great prospective studies must be performed to inform international guidelines.

The strengths of this study include the nested, well-described cohort, the broad assessment of the patients, and the pre-planned follow-up.

However, there are some items to consider. As we failed to investigate CSA in every patient, even in a prospective study, it can be questioned whether such an approach is applicable to other hospitals in practice. On the other hand, a general order to screen for gastric content might improve compliance as quickly as a matter of course, like asking for the last meal and allergies is today.

A key limitation of this study is the low intervention rate; only 29% of eligible participants received CSA, mainly due to organizational barriers in routine clinical practice. This significantly reduced the study’s statistical power and increased the risk of a Type II error. The a posteriori sensitivity analysis underscores the fact that the sample size would need to be larger in order to reliably determine the measured effects.

Nonetheless, the low uptake underscores real-world challenges in implementing such interventions and offers valuable insights for future research.

We were able to include only selected patients who gave informed consent out of the overall cohort at our department. This represents a kind of huge selection bias. On the other hand, we clearly demonstrated the feasibility of a preoperative fasting regimen focused on a narrowly defined cohort. Perioperative fasting concepts do not see everything in black and white. The fasting guideline for each patient may be very individual according to his or her comorbidities, needs, and expectations. Such a concept has been introduced by Rüggeberg et al. with the help of handing out “fasting-cards” in green (for liberal fluid fasting), red (nil per os), and yellow color for an individual concept, as it may be applicable, e.g., for abdominal surgery [17] Our cohort included elderly patients, a scarce entity in the study of Rüggeberg et al. These elderly, multimorbid patients may profit even more from such a measure, as they often are dehydrated even before hospitalization. Especially, a positive effect on neurocognitive disorders may be encountered. A promising area of application for preoperative gastric ultrasound is the large patient group of diabetics. Due to possible gastroparesis, they may experience delayed gastric emptying, which means that the recommended fasting times are not reliable. The use of GLP-1 receptor agonists also exacerbates this problem [15]. Measuring the CSA offers a quick and cost-effective method of increasing patient safety and minimising the risk of aspiration, while at the same time, it is still possible to implement a liberal fluid fasting concept [18].

## 5. Conclusions

In conclusion, liberal fluid fasting was safe even for comorbid elderly patients in trauma and orthopedic surgery. Applying gastric ultrasound may be helpful to increase the safety of patients in a liberal fluid fasting concept, but is dispensable according to the small rate of complications encountered in our study. However, more studies need to confirm our results in other cohorts and settings.

## Figures and Tables

**Table 1:** Demographic and preoperative data.

	Cohort 1 (n = 64)	Cohort 2 (n = 70)	*p*-Value
Age, y (SD)	80.1 (±5.6)	79.8 (±5.7)	0.347
Female sex, n (%)	21 (64)	35 (49)	0.015
BMI, kg m^2^ (IQR)	24.2 (22.3–38.0)	25.5 (22.3–28.2)	0.014
Comorbidities, CCI (IQR)	1 (1–3)	2 (0–5)	0.579
Chronic heart failure, n (%)	8 (13.8)	9 (12.7)	0.465
NYHA class 1, 2, 3, 4; n	2, 15, 9, 1	0, 39, 26, 4	0.996
Hypertension, n (%)	39 (67.2)	51 (71.8)	1
Absolute arrhythmia, n (%)	15 (25.9)	18 (25.4)	1
Diabetes, n(%)	10 (17.2)	9 (12.7)	0.587
Hypothyroidism, n (%)	11 (19.0)	19 (26.8)	0.886
Depression, n (%), GDS (IQR)	5 (8.6), 1 (0–3)	3 (4.2), 1 (0–3)	0.465
Active smoker, n (%)	9 (15.5)	11 (15.5)	0
Pack years	15 (7–50)	15 (0–40)	0.734
Number of medications, n (%)	9 (4–15)	8 (3–10)	0.986
Frailty criteria (Fried), n (%)	2 (1–3)	2 (0–2)	0.368
MoCA	23 (19–26)	24 (20–27)	0.321
Pain, VAS	50 (45–70)	50 (50–70)	0.825
EQ5D-L	9 (7–16)	10 (7–13)	0.945
IADL	7 (0–8)	7.5 (0–8)	0.242
Hearing aids, n (%)	7 (12.1)	4 (5.6)	0.690
Visual aids, n (%)	6 (10.3)	19 (23.8)	0.184
Surgical risk, n (%)			0
Low	29 (50%)	58 (81.7)	
Intermediate	29 (50%)	13 (18.3)	

BMI body mass index, CCI Charlson Comorbidity Index, MoCA Montreal Cognitive Assessment, VAS visual analgesia scale, EQ5D-L EuroQol Group 5D version instrument, GDS Geriatric Depression Scale.

**Table 2:** Pre- and postinterventional measures of gastric content as determined by ultrasound of the antral cross-sectional area (CSA). Values in mean (±Standard deviation), [Range].

	Cohort 1	Cohort 2	*p*
CSA, cm^−1^ (n = 39)	4.6 (±2.1) [1.77–16.2]	5.3 (±3.3) [1.77–16.2]	0.558
Gastric content, mL (n = 39)	10.6 (±16.4) [0–58.2]	11.9 (±19.8) [0–63.2]	0.849
Fluid fasting time span, h	8.84 (±5.53) [0.5–18.0]	1.84 (±1.79) [0–7.0]	<0.0001

**Table 3:** Overview of severe adverse events in cohorts one and two.

	Cohort 1	Cohort 2
Death	0	0
ICU	1	2
Pneumonia	1	0

## Data Availability

The raw data supporting the conclusions of this article will be made available by the authors on request.
